# Prevalence and change in social inequalities in physical activity before and during the COVID-19 pandemic in Sweden

**DOI:** 10.1186/s12939-023-01835-4

**Published:** 2023-01-30

**Authors:** Frida Brattlöf, Per E. Gustafsson, Miguel San Sebastián

**Affiliations:** Department of Epidemiology and Global Health, Umeå University, 901 87 Umeå, Sweden

**Keywords:** Exercise, Socioeconomic factors, COVID-19, Sweden

## Abstract

**Background:**

Physical activity is crucial for our wellbeing. Since the COVID-19 pandemic emerged, physical activity behaviour has changed globally, and social inequalities that already exist in physical activity have increased. However, there is limited knowledge of how these inequalities have evolved in Sweden. Thus, the aim of this study was to assess the prevalence of physical activity, and the socioeconomic inequalities in physical activity before and during the COVID-19 pandemic.

**Methods:**

This study analysed data from the national ‘Health on Equal Terms’ survey which was conducted on participants between 16 and 84, through a repeated cross-sectional design in 2018 (pre-pandemic) and 2021 (during the pandemic). The socioeconomic variables included gender, age, education, occupation, income, and place of birth. For both years, the prevalence of low physical activity, the absolute risk differences, the slope index of inequality, and the slope index difference for each of the variables were calculated.

**Results:**

The level of physical activity increased for the total population studied. However, the social inequalities that existed in 2018 increased over time and across age, education, occupation, income, and place of birth, but not with regard to gender.

**Conclusions:**

Even though the Swedish population increased their levels of physical activity during the COVID-19 pandemic, the social inequalities that already existed in physical activity increased. Interventions to increase the level of physical activity among the young, people with low socioeconomic status, and those born outside Sweden are needed to reduce these social inequalities, and to improve the Swedish population’s wellbeing.

## Introduction

Physical inactivity is responsible for up to 5 million deaths per year globally [1], with an estimated 20 to 30% increased risk of death for people who are not active enough compared to those who are [2]. Despite being a major share of the global burden of disease that is potentially preventable, as much as one in four of the global adult population still do not meet the recommended level of physical activity of 150–300 min of aerobic, moderate-intensity physical activity per week [2].

The coronavirus pandemic (COVID-19) that emerged in 2020, also brought about further challenges for health promotion by physical activity. The lockdown measures implemented by many governments to prevent the spread of the virus [3] may also have had an unintended impact on population health by restricting physical activity behaviour [4]. For example, the opportunities to regular leisure time and commuting physical activity were disrupted by limited gym access [5] and widespread orders to work from home [3], which may have contributed to a more sedentary lifestyle within the confines of the home. Nevertheless, studies have shown inconsistent results regarding the changes in physical activity during the pandemic. For instance, while research from the USA reported an increase in physical activity during the pandemic [6], another study from the United Kingdom observed an increase only among older adults, with a decrease in physical activity among young people [4]. Researchers have also found that changes in physical activity may vary depending on the pandemic wave being studied, with greater reductions in daily physical activity during the first compared to the second wave [7].

In addition, some studies have reflected how social inequalities in physical activity changed during the pandemic. For example, before the pandemic, women in the USA did less exercise than men, and the gender gap increased during the pandemic [6]. In contrast, an analysis of five British cohort studies also presented fewer active women before the pandemic, but displayed a decreased gender inequality during the pandemic [8]. However, another study from the same country did not find an association between gender and change in physical activity during the pandemic [4]. Further studies from the USA have also shown an increased inequality in physical activity between the high and low educated, as well as between high- and low-income earners, as a consequence of the pandemic [6, 9].

In Sweden, one third of the adult population do not reach the recommended level of physical activity, and this is a public health concern [10]. To complicate the picture, the most socially disadvantaged tend to engage in less activity along a social gradient [11]. In response to the COVID-19 pandemic, the authorities in Sweden lacked the legal capacity to enforce compulsory and universal lockdown measures, and instead opted for a less restrictive strategy which relied on voluntary recommendations of social distancing [12]. Despite this comparatively lenient pandemic strategy, the Public Health Agency of Sweden foresaw that the already existing inequalities would be aggravated during the COVID-19 pandemic [13]. However, to our knowledge, only one study has explored social inequalities in physical activity during the pandemic in Sweden; it reported less favourable development of physical activity among women in comparison to men and among blue (manual) compared to white-collar (non-manual) workers during the pandemic [7]. However, the study outcomes were based on perceived change in physical activity without a pre-pandemic measure, increasing the risk of bias. To overcome these shortcomings, and to increase knowledge in this field, the present study aimed to assess the prevalence of physical activity and the socioeconomic inequalities in physical activity before and during the COVID-19 pandemic using two waves of a population-based Swedish national survey.

## Methods

### Study design

A cross-sectional study was carried out based on data from the Health on Equal Terms (*Hälsa på lika villkor*) survey conducted in the years 2018 (pre-pandemic) and 2021 (during pandemic) by the Public Health Agency of Sweden. The survey has been conducted every other year since 2016 as an unbounded random sample of all Swedish residents aged 16–84, with an extra survey performed in 2021 because of the pandemic [14]. The 2020 survey was excluded because it was conducted at the beginning of the pandemic. The sample included individuals between 16 and 84, with a total of 16,756 individuals (42.1% response rate) in 2018 and 17,578 individuals (44.1% response rate) in 2021, respectively [15].

The questionnaire collected information on several self-reported health conditions, health behaviours, and psychosocial and social circumstances. Moreover, through the Swedish Personal Identity Number, the survey data was linked to individual-level register data on age, education, income, occupation, and place of birth from the total population registers of Statistics Sweden (SCB).

### Outcome

The outcome was based on the question “How much time do you spend, during a normal week, on everyday activities, for example walks, biking, or gardening? Add up all the time (at least 10 min at a time)”, explicitly specifying that the question referred to “moderately strenuous physical activity that makes you somewhat heavier than normally, e.g., walking at a brisk pace, gardening, biking, or swimming”. The possible answers included: 0 min/no time, less than 30 min, 30–59 min, 60–89 min, 90–149 min, 150–299 min, and 5 h or more. The outcome was dichotomised into low (0–149 min) and recommended (150 min or more) levels of physical activity. This cut-off point was used based on the WHO’s recommended level of moderate-intensity physical activity of at least 150 min a week [2].

### Socioeconomic variables

Six socioeconomic variables were included in the study: gender, age, education, occupation, income, and place of birth. Gender was divided into male and female and age into four groups: 16–29, 30–44, 45–64, and 65–84. The level of education was classified into three categories: low education (compulsory school), medium education (high school or university programmes of less than three years), and higher education (postgraduate) according to the classification by Statistics Sweden [16]. The variable occupation was divided into four groups based on educational requirement for the occupation: low (no or little education requirement), low–medium (high school competence needed), high–medium (a shorter university programme in addition to high school competence), and high (at least a 3-year university programme) following Statistics Sweden’s classification [17]. Individual disposable income, defined as the amount left for consumption or savings after taxes have been paid, and all positive and negative transfers have been made was divided into five quintiles (quintile 1 being the richest). Place of birth was divided into three groups: Sweden, high-income countries, and low/middle-income countries, based on the World Bank classification of countries according to their gross national income per capita [18].

### Data analysis

First, descriptive statistics were calculated for each variable in 2018 and 2021. The prevalence of low physical activity was then calculated for both years and each of the exposure variables. After that, the absolute risk differences (ARD) for 2018 and 2021, respectively, were estimated through linear-binominal regression. Based on the physical activity prevalence in the present study, the following groups were used as references: female, age 65–84, high education, high occupation, high income, and Sweden as place of birth. The slope index of inequality (SII) was estimated for each variable and each year to examine the degree of socioeconomic inequality in physical activity. The SII is an absolute measure of the social gradient in health that assesses the difference between the most and least advantaged groups, while considering the prevalence and sample size in each category of the socioeconomic variable. A SII of 0 indicates no social gradient in the outcome while, in this study, a value above 0 indicates that the more disadvantaged group has lower physical activity compared to the more advantaged group [19]. Finally, an interaction term between each socioeconomic variable and year (2018 and 2021) was applied, in order to estimate changes in socioeconomic inequalities in physical activity over time (pre- and during the pandemic). To indicate statistical significance, 95% confidence intervals were calculated, and sample weights applied to all analyses. Stata 14.1 statistical software was used for the statistical analysis.

### Ethics

This study was approved by the Swedish Ethical Review Authority (decision no 2021-02398).

## Results

### Population characteristics

Table 1 describes the study population characteristics for 2018 and 2021, and shows similar distribution of the included variables across the two years. Around one third of the participants were between 45 and 64 years old, and the majority had a medium level of education (around 57%). Regarding occupation, nearly half of the participants (about 45%) belonged to the low–medium occupational group, and the majority (80%) were born in Sweden.

**Table 1:** Socioeconomic characteristics of the participants in the ‘Health on equal terms’ surveys

	2018 n (%)^a^	2021 n (%)^a^
Socioeconomic variable		
*Gender*		
Male	8 457 (50.47)	8 887 (50.56)
Female	8 299 (49.53)	8 691 (49.44)
*Age*		
16–29	3 504 (20.91)	3 491 (19.86)
30–44	4 104 (24.49)	4 455 (25.34)
45–64	5 306 (31.67)	5 533 (31.48)
65–84	3 842 (22.93)	4 099 (23.32)
*Education*		
Low education	3 120 (18.82)	3 252 (18.67)
Medium education	9 811 (59.18)	9 710 (55.75)
High education	3 647 (22)	4 455 (25.58)
*Occupation*		
Low	466 (5.16)	417 (4.7)
Low-medium	4 189 (46.35)	3 838 (43.18)
High-medium	1 465 (16.21)	1 379 (15.51)
High	2 918 (32.29)	3 254 (36.61)
*Income*	253,878.1 (347,562.9)	273,977.1 (363,203.2)
*Place of birth*		
Sweden	13 415 (80.07)	13 821 (78.63)
High income countries	1 306 (7.79)	1 292 (7.35)
Low/Middle-income countries	2 034 (12.14)	2 464 (14.02)

^a^For income: *n* = mean, () = standard deviation

### Prevalence

Table 2 displays the prevalence of low physical activity, the crude ARD, the SII for both periods, and the SII difference. A notable reduction of the outcome was seen over time for the total population (from 66% in 2018 to 59% in 2021), and for each of the socioeconomic categories in the study.

**Table 2:** Prevalence, ARD, SII of low physical activity, and SII difference over time

	Prevalence 2018 (%)	Prevalence 2021 (%)	ARD 2018 (95% CI)	ARD 2021 (95% CI)	SII difference 2018–2021 (95% CI)
Socioeconomic variables					
Total	66.01	59.41			
*Gender*					
Female	64.05	57.29	Ref^a^	Ref^a^	
Male	67.94	61.47	3.89 (2.30, 5.48)	4.19 (2.58, 5.79)	
SII			7.77 (4.59, 10.95)	8.37 (5.15, 11.59)	0.60 (-3.92, 5.12)
*Age*					
65–84	65.32	57.02	Ref^a^	Ref^a^	
45–64	65.06	57.11	-0.26 (-2.10, 1.58)	0.09 (-1.79, 1.98)	
30–44	65.57	60.08	0.26 (-1.93, 2.45)	3.06 (0.83, 5.28)	
16–29	68.71	64.97	3.40 (0.94, 5.85)	7.95 (5.47, 10.43)	
SII			3.53 (0.68, 6.38)	9.39 (6.52, 12.27)	5.86 (1.81, 9.91)
*Education*					
High	60.86	52.33	Ref^a^	Ref^a^	
Medium	65.31	59.06	4.45 (2.52, 6.39)	6.74 (4.85, 8.62)	
Low	73.32	69.18	12.47 (10.02, 14.91)	16.85 (14.40, 19.30)	
SII			15.91 (12.76, 19.07)	21.29 (18.16, 24.41)	5.37 (0.93, 9.82)
*Occupation*					
High	59.91	51.91	Ref^a^	Ref^a^	
High-medium	63.30	56.37	3.39 (0.15, 6.62)	4.47 (1.16, 7.78)	
Low-medium	65.15	61.27	5.24 (2.82, 7.66)	9.37 (6.89, 11.84)	
Low	69.99	65.25	10.08 (4.72, 15.43)	13.34 (7.35, 19.34)	
SII			19.49 (11.78, 27.20)	32.29 (24.31, 40.27)	12.80 (1.70, 23.90)
*Income*					
Highest	63.12	52.28	Ref^a^	Ref^a^	
High	61.71	57.88	-1.40 (-4.08, 1.28)	5.60 (3.14, 8.06)	
Medium	64.69	57.88	1.57 (-1.03, 4.17)	5.59 (3.07, 8.12)	
Low	65.76	61.29	2.64 (0.04, 5.25)	9.01 (6.50, 11.52)	
Lowest	71.25	66.12	8.14 (5.61, 10.67)	13.84 (11.36, 16.31)	
SII			11.51 (8.65, 14.37)	15.82 (13.00, 18.64)	4.31 (0.29, 8.33)
*Place of birth*					
Sweden	63.60	56.12	Ref^a^	Ref^a^	
High-income countries	66.71	63.20	3.11 (-0.07, 6.29)	7.08 (3.83, 10.32)	
Low/Middle-income countries	81.42	75.84	17.82 (15.35, 20.29)	19.71 (17.24, 22.19)	
SII			25.36 (21.31, 29.41)	31.33 (27.26, 35.40)	5.97 (0.23, 11.71)

^a^Ref = Reference group

### Socioeconomic inequalities in physical activity in 2018 and 2021

**Fig. 1:**
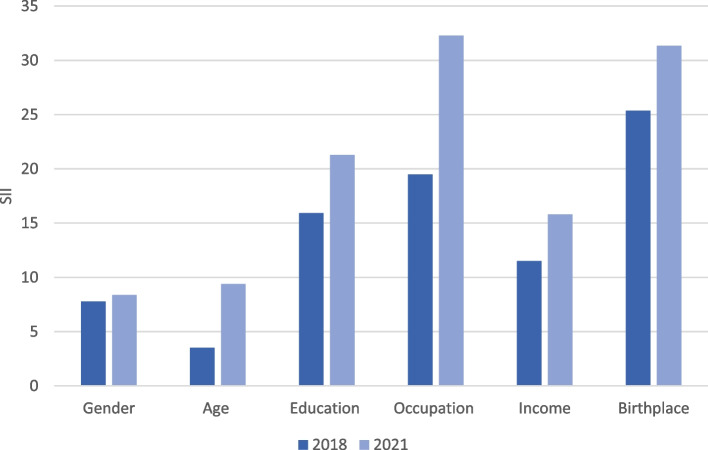
Slope index of inequality (SII) for each social variable by year

As illustrated in Table 2 and Fig. 1, there was a significant social inequality in physical activity in all the studied variables, particularly in education, occupation, income, and place of birth in 2018 and 2021. The largest inequalities were found in place of birth in 2018 (SII: 25.36; 95% CI: 21.31, 29.41) and 2021 (SII: 31.33; 95% CI: 27.26, 35.40) and occupation in 2018 (SII: 19.49; 95% CI: 11.78, 27.20) and 2021 (SII: 32.29; 95% CI: 24.31, 40.27) followed by education in 2018 (SII: 15.91; 95% CI: 12.76, 19.07) and 2021 (SII: 21.29; 95% CI: 18.16, 24.41).

### Socioeconomic inequalities in physical activity over time

With the exception of gender, there was a statistically significant increase in the social inequality in physical activity over time in all social groups. The largest increase was observed in occupation (SII difference: 12.80; 95% CI: 1.70, 23.90) with an approximate increase of 5 percentage points in the remaining significant variables: age (SII difference: 5.86; 95% CI: 1.81, 9.91), education (SII difference: 5.37; 95% CI: 0.93, 9.82), income (SII difference: 4.31; 95% CI: 0.29, 8.33) and place of birth (SII difference: 5.97; 95% CI: 0.23, 11.71).

## Discussion

This study assessed the physical activity prevalence and changes in social inequalities in physical activity before and during the COVID-19 pandemic. The recommended level of physical activity increased between 2018 and 2021 for all the categories that were studied, but the social inequalities persisted. The social inequality in physical activity increased during the pandemic for all the other variables that were studied (age, education, income, occupation, and place of birth) with the exception of gender.

Overall, an increase in physical activity during the pandemic was observed for the total study population and the different social categories. Similarly, several studies from high income countries have reported an increase in physical activity during the pandemic [6, 20]. In contrast, a study from the USA found a decrease in physical activity [21], which could possibly be explained by these studies being carried out at different stages of the pandemic, and with generally stricter pandemic responses than in Sweden. During the early stages, people might have avoided gyms and sporting activities to prevent the viral spread, whereas in later stages, people probably adapted their physical activity behaviour due to immunisation coverage and/or the easiness of the lockdown measures. For instance, this was observed in a Swedish study that indicated greater reductions in daily physical activity during the first pandemic wave (April to June 2020) compared to the second wave (September to December 2020) [7].

In contrast to patterns in other countries [6, 8], in this study, women did more exercise than men in 2018 and 2021, but there was no significant increase in gender inequality over time, which is similar to a study from the UK [4]. In contrast, a Swedish study reported a 38% higher risk of decreased physical activity during the pandemic for women compared to men [7]. Different study designs, time periods, and outcome definitions could explain these differences.

Further, in this study, the elderly (65–84) were more active than younger people (16–29) in both 2018 and 2021, with increasing inequality over time. This finding is similar to a UK study that showed a decrease in physical activity among young people, but an increase among those above 65 [4]. Even though people over 70 were urged by the UK government not to go outside, they managed to increase their level of physical activity [4]. The greater worries about COVID-19 among the young compared to the old were suggested as a possible reason for these findings [4].

Highly educated people with a low level of occupation, low-income earners, and people born in Sweden did more exercise than their counterparts in 2018 and 2021. During the pandemic, the socioeconomic gap in physical activity increased with more socially advantaged groups doing more exercise than the disadvantaged ones. In parallel to these results, studies from the USA have shown an increased inequality in physical activity between the high and low educated, as well as between high- and low-income earners during the pandemic [6, 9]. Even in Sweden, an increased inequality by occupation has been reported [7].

When lockdown measures were introduced, gyms and sports facilities had to limit the number of visitors [5] which made some physical activity less accessible for the population in Sweden. However, in contrast to blue-collar workers, white-collar ones could work from home [3] which decreased commuting time and permitted a flexibility which probably generated more time for physical activity.

We could not find any research investigating place of birth and change in physical activity during the pandemic. However, the low socioeconomic status in Sweden associated with those who are foreign born [22, 23] could explain the increasing inequality found.

### Strengths and limitations

The national representativeness of the study, the presence of two self-reported physical activity measurements over time, the use of register data for certain socioeconomic variables, and the relatively large sample can be considered to be strengths of the study. However, this study has several limitations that should be considered when interpreting the results. The design does not disentangle between any changes in inequities in physical activity caused by the pandemic, pandemic-related restrictions, potential competing interventions, or secular trends, and attribution of the observed changes to pandemic-related causes should therefore be done with caution. Given the self-reported nature of the study design, recall bias, particularly of the outcome, could have been present. The outcome was based on how much time the participants spent each week on everyday activities such as walks, biking, or gardening, and it might have been difficult to remember the exact time spent on each of them. Selection bias could also have been present due to the moderate response rates (42% in 2018 and 44% in 2021), thus challenging the generalisability of the study to the rest of the Swedish population. Further, work-related activities that can generate a certain level of exercise were not included in this study and the outcome focussed only on moderate but no vigorous-intensity physical activity which could have underestimated its prevalence. However, it was not possible to assess the extent of these potential impacts on the results.

## Conclusions

This study, conducted on the Swedish population, has shown an increase in physical activity during the COVID-19 pandemic. However, social gradients that already existed in the level of physical activity increased across age, education, occupation, income, and place of birth, but not with regard to gender. Interventions to increase physical activity among the young, those of low socioeconomic status, and those born outside Sweden should be prioritised in order to improve their wellbeing, and to reduce the social inequalities in physical activity. Special attention should be devoted to preventing increases in these social inequalities in times of pandemics, such as that of COVID-19.

## Data Availability

The data used in this article can be obtained from The Public Health Agency of Sweden. (Folkhälsomyndigheten) on request.
